# Analysis of gaps in rapeseed (*Brassica napus* L.) collections in European genebanks

**DOI:** 10.3389/fpls.2023.1244467

**Published:** 2023-10-09

**Authors:** Stephan Weise, Roel Hoekstra, Kim Jana Kutschan, Markus Oppermann, Rob van Treuren, Ulrike Lohwasser

**Affiliations:** ^1^ Genebank Department, Leibniz Institute of Plant Genetics and Crop Plant Research (IPK) OT Gatersleben, Seeland, Germany; ^2^ Centre for Genetic Resources, the Netherlands (CGN), Wageningen University & Research, Wageningen, Netherlands

**Keywords:** rapeseed, plant genetic resources, gap analysis, niche modelling, *Brassica*

## Abstract

Rapeseed is one of the most important agricultural crops and is used in many ways. Due to the advancing climate crisis, the yield potential of rapeseed is increasingly impaired. In addition to changing environmental conditions, the expansion of cultivated areas also favours the infestation of rapeseed with various pests and pathogens. This results in the need for continuous further development of rapeseed varieties. To this end, the potential of the rapeseed gene pool should be exploited, as the various species included in it contain promising resistance alleles against pests and pathogens. In general, the biodiversity of crops and their wild relatives is increasingly endangered. In order to conserve them and to provide impulses for breeding activities as well, strategies for the conservation of plant genetic resources are necessary. In this study, we investigated to what extent the different species of the rapeseed gene pool are conserved in European genebanks and what gaps exist. In addition, a niche modelling approach was used to investigate how the natural distribution ranges of these species are expected to change by the end of the century, assuming different climate change scenarios. It was found that most species of the rapeseed gene pool are significantly underrepresented in European genebanks, especially regarding representation of the natural distribution areas. The situation is exacerbated by the fact that the natural distributions are expected to change, in some cases significantly, as a result of ongoing climate change. It is therefore necessary to further develop strategies to prevent the loss of wild relatives of rapeseed. Based on the results of the study, as a first step we have proposed a priority list of species that should be targeted for collecting in order to conserve the biodiversity of the rapeseed gene pool in the long term.

## 1 Introduction

Crop plants are major components of human and animal nutrition (Grusak and DellaPenna, 1999), and play an important role as renewable resources or as basic ingredients for chemical or pharmaceutical industry (Metzger and Bornscheuer, 2006; Tilman et al., 2006). Rapeseed or canola (*Brassica napus* L.) is rated amongst the most important agricultural crops. It is used as high-quality edible oil, as high-protein feed for livestock breeding, as biofuel or as raw material for chemical industry like surfactants, softening agents or biodegradable varnishes (Bell, 1982; Piazza and Foglia, 2001; Link, 2008).

Amongst oil plants, rapeseed holds the second largest global market share after soybean (USDA, 2023). In addition, it is also one of the most important sources of protein for animal feed (Hu et al., 2021). Rapeseed provides an average of 40–50% of oil on a dry basis and a protein content of 17–26%. This was achieved by means of breeding programmes starting in the 1970s, which resulted in a significant reduction of harmful glucosinolates and erucic acid (Link, 2008; Jahreis and Schäfer, 2011; Barthet, 2016; Dhillon et al., 2016), in double-low (also called double-zero) cultivars. Rapeseed is the major crop for oilseed production in Europe (Carré and Pouzet, 2014).

Due to the progressing climate crisis, the yield potential of rapeseed is increasingly affected. In particular, rising temperatures, shifting rainfall patterns and increasing incidence of extreme weather events lead to unfavourable effects, such as decreasing yield (Qian et al., 2018). Changed environmental conditions and the extension of acreage foster the infestation of rapeseed with different pests and pathogens (Link, 2008; Williams, 2010). Amongst the most important pathogens are the beet western yellows virus, the *Phoma lingam* and *Verticillium* species, respectively (Gilligan et al., 1980; Heale and Karapapa, 1999; Fitt et al., 2006; Hwang et al., 2016).

In order to cope with the increasing demand for rapeseed and with climatic changes, it is necessary to improve rapeseed varieties continuously. Therefore, it is promising to exploit the potential of its gene pool for breeding through crossing with related species from the primary, secondary and tertiary gene pool, respectively (Chen and Heneen, 1990; Girke et al., 2012). The high potential of crop wild relative (CWR) species was first recognised in the 1920s by the Russian geneticist Nikolay Ivanovich Vavilov (Vavilov, 1926). CWRs usually have a broader genetic variability than crop plants domesticated over hundreds of years (Singh, 2001; McCouch, 2004; Vollbrecht and Sigmon, 2005). The importance of CWRs is continuously growing through scientific progress, e.g. by biotechnological methods enabling gene transfer between distantly related species (Hajjar and Hodgkin, 2007; Ford-Lloyd et al., 2011; Maxted et al., 2012).

In order to simplify CWR classification, Harlan and de Wet proposed to categorise the total available gene pool of a crop and its related species into three groups depending on their degree of relationship with the crop of interest (Harlan and de Wet, 1971). The primary gene pool contains the cultivated species and taxa with which it is completely inter-fertile, thus allowing easy inter-crossing. The secondary gene pool comprises taxa from different species, which are nonetheless closely related. These species can be used for crossing with at least some fertile hybrids. Gene transfer is difficult and may require the use of biotechnological techniques. The tertiary gene pool consists of more distantly related species. Crossing is only possible through the use of biotechnological techniques, such as embryo rescue or bridge crossing, requiring considerable effort.

The gene pool of rapeseed contains species with a large variety of promising resistance alleles against pests and pathogens. Related species, such as *Brassica elongata* Ehrh., *Brassica nigra* (L.) W. D. J. Koch, *Brassica juncea* (L.) Czern., *Sinapis alba* L. or *Sinapis arvensis* L., are known to harbour resistance genes against *P. lingam* whereas *Brassica rapa* L. has been found to show resistances against *Verticillium* wilt (Gerdemann-Knörck et al., 1994; Diederichsen and Sacristan, 1996; Snowdon et al., 2000; Chen et al., 2007; Rygulla et al., 2007; Wei et al., 2010). However, the biological diversity of crop wild relatives is increasingly threatened, not only by the changing climate but also by population expansion, urbanisation and environmental pollution, respectively (Bakarr et al., 2007; Jarvis et al., 2008; van Treuren et al., 2012).

In order to prevent the extinction of species on the one hand and to provide new impulses to breeding programmes on the other hand, it is indispensable to further develop strategies for preserving plant genetic resources for food and agriculture (PGRFA) (Maxted et al., 2012; Parra-Quijano et al., 2012). Important contributions to the preservation of PGRFA are the collection, the maintenance and the characterisation of crop plants and crop wild relatives. In particular, genebanks play an important role for the long-term conservation of PGRFA (Hoisington et al., 1999). There are about 1,800 collections conserving PGRFA around the world. Thereof, about 625 collections are maintained in Europe comprising more than 2 million accessions (Engels and Maggioni, 2012).

A widely accepted goal for the conservation of plant genetic resources is to conserve 95% of all alleles of a random locus that occur in a target population at a frequency of more than 5% (Nagel et al., 2022). However, for decades there has been controversy about what this means in terms of the minimum number of genebank accessions required. When collecting species, there is a tension between the goal of representing the greatest possible genetic diversity of the individual species and the simultaneous practical requirement of having to limit the size of the samples to a manageable level (Allard, 1970). Marshall and Brown (1975) have suggested sampling 50 populations within an ecogeographical region, collecting 50 individual plants per population. In contrast, Crossa et al. (1993) consider that a sample size of 160-210 plants is sufficient. Lawrence et al. (1995), in turn, conclude that 172 randomly sampled plants from a population of a species are sufficient to maintain genetic diversity. When collecting several populations, it is sufficient to take no more than 172 plants per population divided by the number of populations. Irrespective of this discussion, even in the case of a very intensive collection of individual species, the question arises to what extent genetic diversity is covered by a large number of accessions alone (Maxted et al., 2008). For this purpose, other evaluation criteria must also be taken into account, for example the taxonomic composition of the gene pool, the threat status or ecogeographical aspects. It is assumed that sampling populations from distant sites and different habitats will give a more representative coverage of the genetic diversity of a taxon (Maxted et al., 1995). However, most of the existing collections of a target crop hardly contain a representation of the entire known population of the target crop.

In that regard, gap analysis is an important aspect of genetic resources management. In general, gap analysis is a technique to identify shortcomings in biodiversity conservation actions, e.g. missing biodiversity in plant genetic resources collections or in protected areas (Jennings, 2000; Margules and Pressey, 2000; Maxted et al., 2008). It comprises various steps (Burley, 1988): (1) to identify the biodiversity within a region; (2) to examine existing conservation approaches, e.g. protected areas; (3) to determine, which elements of the biodiversity are underrepresented by the existing conservation approaches; (4) to define additional conservation actions.

In principle, gap analysis is applicable to both *ex situ* and *in situ* conservation. The present paper focusses on the *ex situ* conservation of PGRFA in genebank collections. In this context, gap analysis helps to identify the geographical distribution of species of interest and allows comparing with existing genebank holdings. Detected gaps can then be closed, e.g. by organising collecting expeditions.

Comprehensive information about the composition of European germplasm collections is available from the European Search Catalogue for Plant Genetic Resources (EURISCO) (Weise et al., 2017; Kotni et al., 2023). EURISCO is an information system, which documents more than two million accessions maintained *ex situ* in more than 400 collections. It is maintained on behalf of the European Cooperative Programme for Plant Genetic Resources (ECPGR) and is based on a network of National Inventories of 43 member countries from Europe and beyond.

To prioritise species for the improvement of genebank collections, the expected effects of climate change on the distribution of species in their natural environment should be taken into account. In addition, other threat assessments should also be taken into consideration, such as the IUCN Red List of Threatened Species (IUCN, 2023).

Since the expected climate changes depend on a large number of factors, various scenarios have been developed that are referred to as Representative Concentration Pathways (RCPs). Four scenarios have become established (RCP 2.6, RCP 4.5, RCP 6.0 and RCP 8.5), which predict possible changes in greenhouse gas emissions up to the year 2100 in relation to the pre-industrial age around the year 1750 (van Vuuren et al., 2011). This paper uses only the two most contrasting scenarios RCP 2.6 and RCP 8.5 to assess the impacts of climate change. RCP 2.6 is an optimistic scenario assuming that greenhouse gas emissions will decline after 2020. In this case, a global temperature increase of 0.3 to 1.7°C is expected between 2081 and 2100. In contrast, in the pessimistic scenario RCP 8.5, greenhouse gas emissions continue to rise resulting in a temperature increase of 2.6 to 4.8°C over the same period (Stocker et al., 2013). There’s nowadays little doubt that climate change will have significant effects on the distribution of species in their natural habitats, as the current environmental conditions will very likely be affected by climate change. Such effects can be estimated using Species Distribution Models (SDMs). In these models, the presence of a particular species at geographical locations is related to the local environmental conditions, such as temperature and precipitation parameters, after which these relationships can be used to predict species occurrence at other locations. When climate change scenarios are included in the modelling, predictions can be made of the future distribution of a species (Aguirre-Gutiérrez et al., 2017).

Here, we aim to analyse the representation of rapeseed and its wild relatives in European genebank collections and to identify gaps. In addition, ecological niche modelling was used to predict the effects of climate change on future species distributions in order to prioritise relevant species for conservation.

## 2 Materials and methods

### 2.1 Species data

To determine the composition of the primary, secondary and tertiary gene pool of rapeseed, the Crop Wild Relative Inventory (Vincent et al., 2013; CWR, 2023) of the Global Crop Diversity Trust was used. According to the Crop Wild Relative Inventory, the gene pool of rapeseed comprises various taxa from 16 genera. For improving the accuracy of further comparisons with data on germplasm holdings, these taxa were checked for synonym names based on The World Flora Online (WFO, 2023), which resulted in about 900 additional names (including subtaxa). Based on the identified composition of the rapeseed gene pool, germplasm collections across Europe were examined for gaps, i.e. countries in the distribution range of a species for which no accession are available in PGR collections. The emphasis was on European collections, because rapeseed originated from natural crossings of *B. rapa* and *Brassica oleracea* L (Neuffer, 2001). both occurring in the Mediterranean area. Also, the other species of the rapeseed gene pool mainly occur in European temperate areas. Therefore, the natural occurrence ranges of the species of the rapeseed gene pool were determined using the Euro+Med PlantBase (Euro+Med, 2023) and GRIN Taxonomy (GRIN, 2023). Information about the origin countries of genebank accessions, the countries maintaining the accessions as well as the numbers of available accessions was extracted from EURISCO (EURISCO, 2023). This data was then compared with the natural ranges of the species. In addition, the species of the rapeseed genepool were checked against the IUCN Red List of Threatened Species (IUCN, 2023).

### 2.2 Species distribution modelling

Species distribution modelling (or ecological niche modelling) was used to predict the effects of climate change on the future distribution of the wild relatives of *B. napus* in Europe and countries bordering the Mediterranean Sea. Modelling procedures followed the methods described by Aguirre-Gutiérrez et al. (2017) and van Treuren et al. (van Treuren et al., 2017; van Treuren et al., 2020). **Table 1** lists 51 taxa related to rapeseed (*B. napus*). For the modelling, geographic occurrence data of these species were downloaded from the Global Biodiversity Information Facility (GBIF) (GBIF, 2023), with the exception of the five cultivated species (*Brassica carinata* A. Braun, *B. juncea*, *B. napus*, *B. oleracea* and *Raphanus sativus* L.) where it is impossible to distinguish natural occurrences from escapes from cultivation. Five taxa (*Crambe hispanica* subsp. *abyssinica* (Hochst. ex R.E.Fr.) Prina, *Erucastrum abyssinicum* R. E. Fr., *Orychophragmus violaceus* (L.) O.E. Schulz, *Physaria fendleri* (A. Gray) OKane & Al-Shehbaz and *Rorippa indica* (L.) Hiern) had no occurrence data within the studied region. From five taxa (*Brassica dimorpha* Coss. & Durieu, *Brassica deserti* Danin & Hedge, *Brassica desnottesii* Emb. & Maire, *Brassica souliei* Batt. subsp. *souliei* Batt. and *Brassica hilarionis* Post) the number of georeferenced locality data was insufficient for distribution modelling. *Brassica souliei* Batt. (exluding subsp. *amplexicaulis*) was used instead of *Brassica souliei* Batt. subsp. *souliei* Batt. Occurrence data of *Hirschfeldia incana* (L.) Lagr.-Foss. were downloaded using its synonym *Sinapis incana* L. Records from outside the studied region as well as records with missing or incorrect geographic information were removed. For nine taxa having low numbers of occurrences some additional records could be georeferenced using the locality descriptions and Google Earth. A spatial resolution, corresponding to a grid size of 2.5 min of a degree of longitude and latitude in the WorldClim dataset (Hijmans et al., 2005), was used to process the occurrence data. Multiple occurrence data per grid cell were reduced to one observation. To avoid spatial autocorrelation, only records separated by at least one grid cell were used for the distribution modelling, using seven bioclimatic variables (related to temperature and precipitation) and two soil variables (van Treuren et al., 2020). In the **supplementary data**, the downloads from GBIF and the number of grid cells used are given for each taxon^1^. The manually georeferenced records have been made available as well. The R programming language (R_Core_Team, 2019) was used for distribution modelling with the Biomod2 package (Thuiller et al., 2009). Details of the modelling procedures are provided by Aguirre-Gutiérrez et al. (2017) and van Treuren et al. (2017). Predicted occurrences are solely based on the expected suitability of geographic locations as a result of the examined bioclimatic and soil variables. Other factors that may influence species occurrence, such as dispersal ability or geographic barriers, are not taken into account.

**Table 1:** Genepool of *Brassica napus* L. and its representation in European genebanks based on EURISCO data.

Taxon	Accs. in European collections	Countries of native occurrences	Collected from native countries	IUCN Red List category
Primary gene pool				
*Brassica napus* L.	5,922	cultivated	0	–
Secondary gene pool				
*Brassica cretica* Lam.	2,399	Greece, Turkey	97	Least concern
*Brassica juncea* (L.) Czern.	2,479	cultivated	0	–
*Brassica rapa* L.	4,941	Belarus, Bosnia and Herzegovina, Bulgaria, Croatia, Estonia, France, Greece, Hungary, Ireland, Italy, Latvia, Lithuania, Malta, Morocco, Netherlands, Norway, Poland, Romania, Slovakia, Slovenia, Spain, Switzerland, Ukraine, United Kingdom	1,231	Data deficient
*Erucastrum gallicum* (Willd.) O. E. Schulz	28	Albania, Austria, Croatia, France, Italy, Netherlands, Slovenia, Spain, Switzerland	4	–
Tertiary gene pool				
*Brassica bourgeaui* (Webb ex Christ) Kuntze	4	Spain*	4	–
*Brassica carinata* A. Braun	386	naturalised in Ethiopia; cultivated in Africa and Northern America*	287	–
*Brassica deserti* Danin & Hedge	0	Egypt	0	–
*Brassica desnottesii* Emb. & Maire	2	Morocco	1	–
*Brassica dimorpha* Coss. & Durieu	1	Algeria, Tunisia	1	–
*Brassica elongata* Ehrh.	14	Armenia, Austria, Bosnia and Herzegovina, Bulgaria, Croatia, Czech Republic, Hungary, Morocco, Romania, Russian Federation, Serbia, Slovakia, Slovenia, Spain, North Macedonia, Turkey, Ukraine	4	Least concern
*Brassica fruticulosa* Cirillo	42	Algeria, Bosnia and Herzegovina, France, Italy, Malta, Morocco, Spain, Tunisia	26	Least concern
*Brassica gravinae* Ten.	7	Algeria, Italy, Libya, Morocco, Tunisia	3	Data deficient
*Brassica hilarionis* Post	5	Cyprus	3	Endangered
*Brassica incana* Ten.	48	Albania, Bosnia and Herzegovina, Croatia, Greece, Italy, Malta	39	Data deficient
*Brassica insularis* Moris	31	Algeria, France, Italy, Malta, Tunisia	31	Near threatened
*Brassica maurorum* Durieu	7	Algeria, Morocco	5	–
*Brassica montana* Pourr.	59	France, Italy, Spain	46	Least concern
*Brassica nigra* (L.) W. D. J. Koch	415	Belgium, Croatia, Cyprus, Egypt, Israel, Italy, Lebanon, Luxembourg, Montenegro, Netherlands, Spain, Syria	94	Least concern
*Brassica oleracea* L.	11,663	France, Germany, Italy, Spain, United Kingdom	3,173	Data deficient
*Brassica repanda* (Willd.) DC.	29	Algeria, France, Italy, Morocco, Spain, Switzerland	27	Least concern
*Brassica souliei* Batt. subsp. *souliei* Batt.	4	Algeria, Morocco, Tunisia	1	Data deficient
*Brassica souliei* Batt. subsp. *amplexicaulis* (Desf.) Greuter & Burdet	4	Italy, Malta, Morocco	2	Data deficient
*Brassica tournefortii* Gouan	126	Algeria, Cyprus, Egypt, Greece, Israel, Italy, Lebanon, Libya, Portugal, Spain, Syria, Morocco, Malta, Tunisia, Turkey	111	Least concern
*Capsella bursa-pastoris* (L.) Medik.	106	Albania, Algeria, Andorra, Armenia, Austria, Belarus, Belgium, Bosnia and Herzegovina, Bulgaria, Croatia, Cyprus, Czech Republic, Denmark, Egypt, Estonia, Finland, France, Germany, Greece, Hungary, Iceland, Ireland, Israel, Italy, Latvia, Libya, Liechtenstein, Lithuania, Luxembourg, Malta, Moldova, Montenegro, Morocco, Netherlands, North Macedonia, Norway, Poland, Portugal, Romania, Russian Federation, Serbia, Slovenia, Spain, Sweden, Switzerland, Tunisia, Turkey, Ukraine, United Kingdom	93	Least concern
*Crambe hispanica* subsp. *abyssinica* (Hochst. ex R.E.Fr.) Prina	169	Ethiopia, Kenya, Rwanda, Tanzania, Uganda, Democratic Republic of the Congo	2	Least concern
*Descurainia sophia* (L.) Webb ex Prantl	22	Albania, Algeria, Andorra, Armenia, Austria, Belarus, Bosnia and Herzegovina, Bulgaria, Croatia, Czech Republic, Denmark, Egypt, Estonia, France, North Macedonia, Greece, Hungary, Iceland, Israel, Italy, Latvia, Liechtenstein, Lithuania, Malta, Moldova, Montenegro, Morocco, Netherlands, Portugal, Russian Federation, Serbia, Slovenia, Spain, Sweden, Switzerland, Turkey, Ukraine, United Kingdom	6	–
*Diplotaxis acris* (Forsk.) Boiss.	21	Egypt, Israel, Turkey	20	–
*Diplotaxis catholica* (L.) DC.	13	Morocco, Portugal, Spain	13	Least concern
*Diplotaxis erucoides* (L.) DC.	49	Algeria, Egypt, France, Israel, Italy, Lebanon, Malta, Morocco, Portugal, Romania, Spain, Syria, Tunisia, Turkey	48	Least concern
*Diplotaxis harra* (Forssk.) Boiss.	27	Algeria, Egypt, Israel, Italy, Lebanon, Libya, Morocco, Spain, Syria, Tunisia	23	Least concern
*Diplotaxis muralis* (L.) DC.	18	Albania, Algeria, Austria, Belgium, Bosnia and Herzegovina, Bulgaria, Croatia, Czech Republic, Egypt, France, Greece, Hungary, Italy, Libya, Luxembourg, Malta, Moldova, Montenegro, Morocco, Netherlands, North Macedonia, Portugal, Romania, Russian Federation, Serbia, Slovenia, Spain, Switzerland, Tunisia, Turkey, Ukraine	12	Least concern
*Diplotaxis siifolia* Kunze	14	Algeria, Morocco, Portugal, Spain	14	Near threatened
*Diplotaxis tenuifolia* (L.) DC.	27	Albania, Andorra, Austria, Belgium, Bosnia and Herzegovina, Bulgaria, Croatia, Czech Republic, France, Greece, Hungary, Italy, Liechtenstein, Luxembourg, Malta, Moldova, Montenegro, Netherlands, North Macedonia, Portugal, Romania, Serbia, Slovakia, Slovenia, Spain, Switzerland, Turkey, Ukraine	18	Least concern
*Diplotaxis viminea* (L.) DC.	4	Albania, Algeria, Bulgaria, Croatia, Cyprus, Egypt, France, Greece, Israel, Italy, Malta, Morocco, Portugal, Romania, Spain, Tunisia, Turkey, Ukraine	3	Least concern
*Enarthrocarpus lyratus* (Forssk.) DC.	2	Egypt, Jordan	0	–
*Eruca vesicaria* (L.) Cav.	169	Algeria, Bulgaria, Croatia, Egypt, France, Greece, Hungary, Israel, Italy, Lebanon, Libya, Malta, Moldova, Morocco, Portugal, Romania, Spain, Switzerland, Syria, Tunisia, Turkey, Ukraine	110	Least concern
*Erucastrum abyssinicum* R. E. Fr.	2	Eritrea, Ethiopia, Yemen	0	–
*Hirschfeldia incana* (L.) Lagr.-Foss.	131	Albania, Algeria, Andorra, Armenia, Croatia, Cyprus, France, Greece, Israel, Italy, Lebanon, Malta, Morocco, Portugal, Spain, Syria, Tunisia, Turkey, Ukraine	111	–
*Moricandia arvensis* (L.) DC.	15	Algeria, Croatia, France, Greece, Italy, Malta, Montenegro, Morocco, Portugal, Spain, Tunisia	13	–
*Moricandia nitens* (Viv.) E. A. Durand & Barratte	15	Egypt, Israel, Jordan, Libya, Morocco, Tunisia	15	–
*Orychophragmus violaceus* (L.) O.E. Schulz	1	China, Korea	0	–
*Physaria fendleri* (A. Gray) OKane & Al-Shehbaz	0	Mexico, USA	0	–
*Raphanus raphanistrum* L.	236	Albania, Algeria, Armenia, Austria, Belarus, Belgium, Bosnia and Herzegovina, Bulgaria, Croatia, Cyprus, Czech Republic, Denmark, Egypt, Estonia, France, Greece, Hungary, Iceland, Ireland, Israel, Italy, Latvia, Lebanon, Libya, Liechtenstein, Lithuania, Luxembourg, Malta, Moldova, Montenegro, Morocco, Netherlands, North Macedonia, Portugal, Romania, Russian Federation, Serbia, Slovakia, Slovenia, Spain, Sweden, Switzerland, Syria, Tunisia, Turkey, Ukraine, United Kingdom	189	Least concern
*Raphanus sativus* L.	3,550	Cyprus, Israel, Portugal, Spain	240	–
*Rapistrum rugosum* (L.) All.	30	Albania, Algeria, Andorra, Armenia, Austria, Bulgaria, Croatia, Cyprus, Egypt, France, Greece, Israel, Italy, Lebanon, Libya, Malta, Montenegro, Morocco, North Macedonia, Portugal, Russian Federation, Slovenia, Spain, Syria, Tunisia, Turkey, Ukraine	27	–
*Rorippa indica* (L.) Hiern	5	Egypt	0	–
*Rorippa islandica* (Oeder) Borb	8	Armenia, Austria, Bosnia and Herzegovina, Croatia, France, Greece, Iceland, Ireland, Italy, Liechtenstein, Montenegro, North Macedonia, Norway, Russian Federation, Slovenia, Spain, Switzerland, Turkey, Ukraine, United Kingdom	7	Least concern
*Sinapis alba* L.	1,372	Albania, Algeria, Belgium, Bulgaria, Croatia, Cyprus, Denmark, Egypt, France, Germany, Greece, Hungary, Israel, Italy, Lebanon, Libya, Luxembourg, Malta, Montenegro, Moldova, Morocco, Netherlands, Norway, Poland, Portugal, Romania, Spain, Sweden, Switzerland, Syria, Tunisia, Turkey, Ukraine, United Kingdom	739	Least concern
*Sinapis arvensis* L.	144	Albania, Algeria, Austria, Armenia, Belarus, Belgium, Bosnia and Herzegovina, Bulgaria, Croatia, Cyprus, Egypt, Estonia, France, Greece, Hungary, Israel, Italy, Latvia, Libya, Lithuania, Luxembourg, Malta, Moldova, Montenegro, Morocco, Netherlands, Portugal, Serbia, Slovenia, Spain, Russian Federation, Tunisia, Turkey, Ukraine	99	Least concern
*Sinapis pubescens* L.	11	Albania, France, Italy, Algeria, Tunisia	9	Least concern

The countries of native occurrences were manually checked using the Euro+Med PlantBase. Entries with an asterisk (*) were complemented with data from GRIN Taxonomy.

## 3 Results and discussion

Europe and especially the Mediterranean area provide a great richness of species. Coincidently, the most critical collection gaps are related to this area (Castañeda-Álvarez et al., 2016). The Cruciferous (Brassicaceae) family currently comprises 338 genera and 3,709 species (Al-Shehbaz et al., 2006). Thereof, 39 accepted species names belong to the genus *Brassica* (Warwick and Francis, 2006). This number considerably increases when taking into account the large number of existing synonyms. The origins of the *Brassica* species are the area of the Mediterranean and southwest Asia (Al-Shehbaz et al., 2006). Many of the species of the genus *Brassica* are economically important, especially *B. rapa*, *B. nigra*, *B. oleracea*, *B. juncea*, *B. napus* and *B. carinata* (Cheng et al., 2014). This is also reflected by the number of holdings in European germplasm collections. The majority of the *Brassica* species are wild relatives which are not economically significant and therefore collected to a much lesser extent. However, they are of great importance for resistance to abiotic and biotic stresses and have the potential to improve resilience in modern cultivars (Quezada-Martinez et al., 2021). In addition, some of the wild *Brassicas* could even serve as a source of a new crop (Razzaq et al., 2021).

### 3.1 Data basis used

The present study is based on freely accessible data. The main basis is passport data from the EURISCO system, which provides comprehensive data on the majority of European genebank collections. EURISCO is an aggregator database that is unique in terms of the quantity and quality of data available and the underlying network. Nevertheless, there are limitations that need to be considered. Collections of plant genetic resources are sometimes very old and documented to varying degrees. Therefore, it cannot be assumed that there is complete information about the countries of origin and the sites where the genebank samples were found. For this reason, only those accessions could be considered for which collecting information is available. Despite all limitations, this still represents the best possible data available.

Furthermore, data on natural occurrence countries of the different *Brassica* species were used. These data are not necessarily complete either, as they depend heavily on the available literature sources. The fact that a country, in contrast to its immediate neighbours, is not listed as a natural area of origin does not necessarily mean that the corresponding material does not exist there, but only that it has not been described there so far. As mentioned above, an attempt was made to complement data from several sources.

### 3.2 Gene pool inventory and representation in genebanks for rapeseed

Results of the inventory are shown in **Table 1**. The rapeseed gene pool comprises 51 species, of which a total of 34,777 accessions are included in EURISCO. Subtaxa were not considered, as corresponding information is only available for a part of the accessions. No accessions of *B. deserti* and *P. fendleri* were found in European genebank collections. If the inventory is further restricted to accessions originating from the native occurrence areas and marked as collected material, the total number of accessions is 7,001. In this context, only areas known for native occurrences (represented by countries) were considered. Areas, in which those species were introduced or are being cultivated, were ignored.

The primary gene pool of rapeseed only consists of the cultivated species *B. napus*, which is represented by 5,922 accessions in European genebanks. These numbers include not only oil types but also 564 swede accessions (*B. napus* var. *napobrassica* (L.) Rchb.) as well as 65 Siberian kale accessions (*B. napus* var. *pabularia* (DC.) Rchb.).

The secondary gene pool comprises four species, including 9,847 accessions in total. *B. juncea* is a cultivated species, for which 2,479 accessions are preserved. With 2,399 accessions, *Brassica cretica* Lam. is represented with high numbers. However, only 97 of them were collected in native occurrence countries, corresponding to 4% of the total number of accessions of this species. For *Erucastrum gallicum* (Willd.) O. E. Schulz, only 28 accessions are preserved in European genebanks with four of them collected from native occurrence countries. In the case of *B. rapa*, which is partially cultivated, 1,231 (25%) out of 4,941 accessions were collected from native countries.

**Figure 1:**
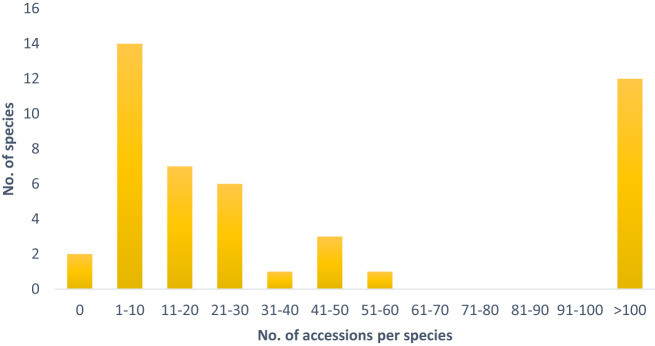
Number of accessions per species of the tertiary gene pool, which are maintained in European germplasm collections.

The tertiary gene pool comprises 46 species, which are represented by 19,008 accessions. Despite some highly represented species, such as the economically important *B. oleracea* (11,663 accessions), *B. nigra* (415 acc.), *B. carinata* (386 acc.) as well as *R. sativus* (3,550 acc.) and *S. alba* (1,372 acc.), the majority of species are only maintained in relatively low numbers in European genebanks (**Table 1**). 35% of the species of the tertiary gene pool are represented by less than 10 accessions and 50% by less than 20 accessions (**Figure 1**).

Even if the natural regions of origin are not considered, it is obvious that the majority of species of the *Brassica* gene pool are underrepresented in European genebanks although collecting missions were carried out in the 1970s (Razzaq et al., 2021). To solve this problem, a global strategy for the conservation of *Brassica* genetic resources was developed by the Global Crop Diversity Trust (Allender and Giovannini, 2023), confirming the importance of CWRs. Countries such as Italy have recognised the importance of *Brassica* CWRs and have established action plans (Ciancaleoni et al., 2021; Perrino and Wagensommer, 2022). Further collecting strategies for the whole of Europe need to be developed to improve *ex situ* conservation of at least some underrepresented species like *E. gallicum* from the secondary genepool which has only four accessions from native countries but nine countries where the species naturally occurs. The CWRs are also important for other *Brassica* genepools beside rapeseed because of the close evolutionary relationship. Rapeseed originated from an interspecific hybridisation of *B. rapa* and *B. oleracea* (Quezada-Martinez et al., 2021).

### 3.3 Conservation status

The Sampled Red List Index shows that approximately 22% of the plant species are threatened with extinction (Brummitt et al., 2015). Out of 1,350 European plant species, more than half are expected to be vulnerable or threatened by 2080 (Thuiller et al., 2005).

The species of the rapeseed gene pool were investigated for their conservation status. According to the IUCN Red List of Threatened Species, *B. hilarionis* is classified as endangered, while *Brassica insularis* Moris and *Diplotaxis siifolia* Kunze are classified as near threatened. With 5, 31 and 14 accessions, respectively, these species are maintained in low numbers in European collections. This refers only to the total accession numbers (34,777, see above); the natural occurrence countries are not taken into account here either. If these are additionally included in the considerations, the underrepresentation is further aggravated.

In general, the numbers presented in **Table 1** indicate that rapeseed gene pool accessions collected at native occurrence countries of the respective species are underrepresented in European genebanks. This situation is even worse when looking at the representation of individual countries belonging to the natural distribution area (**Supplementary Table 1**). For example, the near threatened species, *B. insularis* natively occurs in five different countries, but was only collected in three of them. The near threatened species *D. siifolia* was collected in three out of four countries only, which is a cause for concern given the low number of accessions. *B. hilarionis* (endangered) is endemic to Cyprus and occurs nowhere else. Underrepresentation was also evident for species that currently are of least concern. For *B. elongata*, 17 countries were identified for native occurrences, but only in three of them accessions were collected. For *Descurainia sophia* (L.) Webb ex Prantl, 38 countries were identified, but accessions were only collected in six.

It should be noted here that the data situation regarding the endangerment status only allows limited statements to be made. Most species of the *Brassica* genepool are not endangered or near threatened. 21 out of the 51 species are assigned to the IUCN Red List category of least concern. For six other species, data is indicated as deficient, while 21 species are not listed in the Red List at all. Only three species are endangered or near threatened, respectively. Nevertheless, it is important to improve the future conservation, as they play a major role in crop improvement (Raggi et al., 2022). In this context, of course, conservation under *in situ* conditions should not be ignored, especially for wild species. However, it must be taken into account that survival under *in situ* conditions is by no means guaranteed (e.g. due to climate change). In addition, access to *in situ* material is also difficult for users. A strong focus on *ex situ* conservation is therefore indispensable. Appropriate strategies need to be developed for this. Therefore, as a first step, we propose a priority list for the targeted collecting (see 3.5.)

### 3.4 Effects of climate change on species distribution

**Figure 2:**
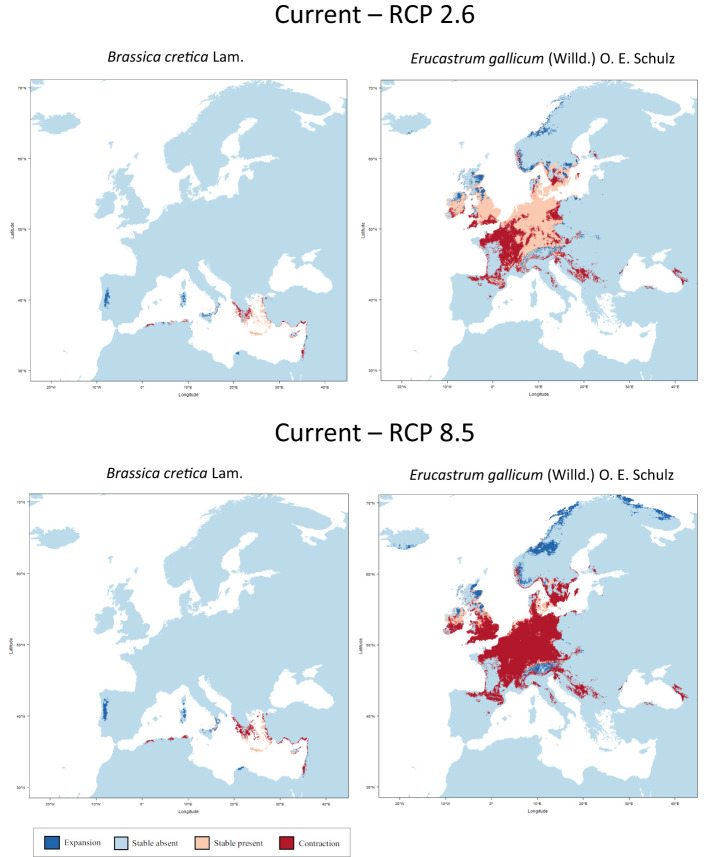
Predicted areas of distribution for the species of the secondary gene pool *B. cretica* and *E. gallicum* under the climate change scenarios RCP 2.6 (optimistic) and RCP 8.5 (pessimistic) for the year 2070 as compared to current conditions.

**Figure 2** shows the results of the niche modelling for the wild species of the secondary gene pool. *B. juncea*. was not considered because it is a cultivated species. *B. rapa* is not shown in **Figure 2** since it is partly cultivated. In the case of *B. cretica* the simulation results show that the range remains almost constant at RCP 2.6 (expansion of 0.03%), but shrinks by 29.4% at RCP 8.5, assuming full migration potential. If there is no migration, however, the range will decrease by 33.9% (RCP 2.6) or 63.7% (RCP 8.5). Even more dramatic are the changes in *E. gallicum* (reduction of 29.5% and 71.5% respectively with migration; reduction of 40.6% and 89.4% respectively without migration) and *B. rapa* (reduction of 9.6% and 26.3% respectively with migration; reduction of 26.2% and 42.6% respectively without migration) (**Table 2**).

**Table 2:** Predicted changes of the distribution area of the species of the secondary and tertiary gene pool in 2070 under RCP 2.6 (optimistic scenario) and RCP 8.5 (pessimistic scenario), both with and without migration.

Taxon	Current range size	Range change with migration		Range change no migration	
		RCP 2.6	RCP 8.5	RCP 2.6	RCP 8.5
Secondary gene pool					
*Brassica cretica* Lam.	6,769	0.03%	-29.4%	-33.9%	-63.7%
*Brassica juncea* (L.) Czern.		cultivated			
*Brassica rapa* L.	250,077	-9.6%	-26.3%	-26.2%	-42.6%
*Erucastrum gallicum* (Willd.) O. E. Schulz	103,289	-29.5%	-71.5%	-40.6%	-89.4%
Tertiary gene pool					
*Brassica bourgeaui* (Webb ex Christ) Kuntze	246	-5.3	-9.3	-5.3	-9.3
*Brassica carinata* A. Braun		Outside study area			
*Brassica deserti* Danin & Hedge		Only 3 GBIF records (2 after cleaning)			
*Brassica desnottesii* Emb. & Maire		Only 5 GBIF records (2 after cleaning)			
*Brassica dimorpha* Coss. & Durieu		Only 1 GBIF record (1 after cleaning)			
*Brassica elongata* Ehrh.	164,251	-15.4	-39.6	-38.4	-72.3
*Brassica fruticulosa* Cirillo	34,699	4.8	-4.6	-27.7	-60.4
*Brassica gravinae* Ten.	62,615	-10.3	-28.3	-31.4	-69.6
*Brassica hilarionis* Post		Only 5 GBIF records (3 after cleaning)			
*Brassica incana* Ten.	3,167	80.2	244.7	-19.1	-49.3
*Brassica insularis* Moris	9,277	-66.0	-78.0	-66.5	-89.0
*Brassica maurorum* Durieu	10,732	-36.9	-62.3	-41.1	-68.3
*Brassica montana* Pourr.	23,133	40.8	23.4	-38.7	-76.0
*Brassica nigra* (L.) W. D. J. Koch	159,081	-0.1	-5.8	-9.0	-26.1
*Brassica oleracea* L.		Cultivated			
*Brassica repanda* (Willd.) DC.	43,996	-46.2	-87.6	-50.8	-89.7
*Brassica souliei* Batt. subsp. *souliei* Batt.	32,540	-45.7	-80.8	-54.1	-85.8
*Brassica souliei* Batt. subsp. *amplexicaulis* (Desf.) Greuter & Burdet	5,685	-77.7	-96.7	-82.1	-97.5
*Brassica tournefortii* Gouan	73,804	-1.8	22.3	-21.9	-23.3
*Capsella bursa-pastoris* (L.) Medik.	280,915	-7.6	-24.4	-16.1	-32.9
*Crambe hispanica* subsp. *abyssinica* (Hochst. ex R.E.Fr.) Prina		Outside study area			
*Descurainia sophia* (L.) Webb ex Prantl	180,923	-17.5	-44.0	-34.6	-66.3
*Diplotaxis acris* (Forsk.) Boiss.	10,445	56.8	133.2	-23.2	-15.5
*Diplotaxis catholica* (L.) DC.	42,412	-6.1	-24.3	-17.7	-47.0
*Diplotaxis erucoides* (L.) DC.	68,478	49.2	37.7	-12.8	-36.1
*Diplotaxis harra* (Forssk.) Boiss.	47,471	39.1	84.0	-7.1	-4.9
*Diplotaxis muralis* (L.) DC.	132,933	2.3	-9.7	-12.9	-35.4
*Diplotaxis siifolia* Kunze	27,283	-17.2	-41.3	-28.7	-52.9
*Diplotaxis tenuifolia* (L.) DC.	127,278	-1.7	-14.5	-16.5	-42.9
*Diplotaxis viminea* (L.) DC.	77,775	29.3	19.1	-9.6	-28.6
*Enarthrocarpus lyratus* (Forssk.) DC.	2,013	-13.3	-16.7	-51.1	-52.9
*Eruca vesicaria* (L.) Cav.	133,801	-1.8	-19.4	-16.8	-38.5
*Erucastrum abyssinicum* R. E. Fr.		Outside study area			
*Hirschfeldia incana* (L.) Lagr.-Foss.	159,233	9.4	-1.6	-8.1	-24.3
*Moricandia arvensis* (L.) DC.	60,456	8.6	17.9	-19.8	-41.8
*Moricandia nitens* (Viv.) E. A. Durand & Barratte	25,965	-6.6	10.2	-29.9	-29.0
*Orychophragmus violaceus* (L.) O.E. Schulz		Outside study area			
*Physaria fendleri* (A. Gray) OKane & Al-Shehbaz		Outside study area			
*Raphanus raphanistrum* L.	255,467	2.6	1.3	-16.1	-24.6
*Raphanus sativus* L.		Cultivated			
*Rapistrum rugosum* (L.) All.	171,637	4.2	-5.9	-8.3	-24.5
*Rorippa indica* (L.) Hiern		Outside study area			
*Rorippa islandica* (Oeder) Borb	108,211	-31.8	-72.5	-41.1	-85.0
*Sinapis alba* L.	203,160	2.3	-0.3	-9.9	-24.0
*Sinapis arvensis* L.	299,489	-8.8	-25.3	-19.6	-36.1
*Sinapis pubescens* L.	38,339	-9.1	9.3	-24.1	-49.3

The current range sizes are given in numbers of cells (~4x4 km). Range changes are presented in percentage.

**Figure 3:**
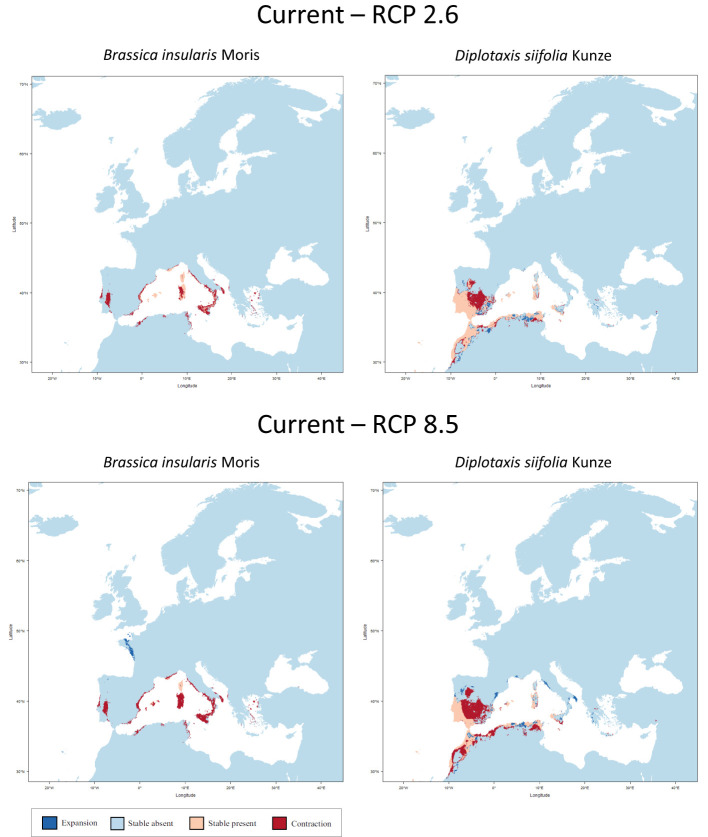
Predicted areas of distribution for Red List species of the tertiary gene pool under the two climate change scenarios RCP 2.6 (optimistic) and RCP 8.5 (pessimistic) for the year 2070 as compared to current conditions. *B. hilarionis* was omitted from the calculations due to insufficient occurrence data.

At this point, the three species listed in the IUCN Red List of Threatened Species as endangered or near threatened deserve closer examination. All three belong to the tertiary gene pool. For *B. hilarionis*, no niche modelling could be performed because the available occurrence data was insufficient. For *B. insularis*, the calculations indicate that the distribution area will decrease by 66.0% (RCP 2.6) or 78.0% (RCP 8.5) assuming full migration potential. Without migration, the decline will even be 66.5% (RCP 2.6) and 89.0% (RCP 8.5), respectively. In the case of *D. siifolia*, a significant reduction of the distribution area is also to be expected (reduction of 17.2% and 41.3% respectively with migration; reduction of 28.7% and 52.9% respectively without migration) (**Figure 3**). This is particularly dramatic against the background of the rather low number of germplasm accessions of these species in European collections. Modelling results of all species of the tertiary gene pool are shown in **Table 2**. In addition to the figures given there, the **Supplementary Data** also shows the predicted changes in the distribution areas for each species on maps.

Niche modelling shows the expected loss of distribution, but also the potential for new areas of occurrence. However, the dispersal ability of species or geographic barriers that restrict migration are not considered. For the studied region, it is not known whether the species are able to reach the areas where favourable climatic conditions prevail.

However, against the background of climate change and its likely effects in the whole of Europe, there is an urgent need for action, which was made evident by the niche modelling carried out. In this context, it should be noted that the modelling results of the optimistic scenario (RCP 2.6) are probably less likely compared to those of the pessimistic scenario (RCP 8.5). The long-term effects of global warming, which is taking place in the 21st century, will also have an impact on the following centuries (Schwalm et al., 2020; Lyon et al., 2022). The niche modelling used here offers the opportunity to react in advance to upcoming changes and to adjust the collection development of the European genebanks accordingly. Based on the results of niche modelling, the development of a collecting strategy for the endangered as well as underrepresented species is therefore urgently needed.

### 3.5 Implications for conservation

As described in the previous sections, the majority of species of the rapeseed gene pool are underrepresented in European genebanks. This concerns in particular CWRs from the secondary and tertiary gene pool, which are also important for other *Brassica* species besides rapeseed due to the close evolutionary relationship between *B. napus*, *B. rapa* and *B. oleracea*. In addition, the CWRs have great potential for future crop improvement.

Based on the composition of the rapeseed gene pool, the information on the IUCN Red List status, the representation of the species in European genebanks (including consideration of the natural countries of origin) and the predicted effects of climate change on the future distribution ranges, we would like to propose a priority list for targeted collection. For this purpose, the species were assigned to three priority groups (high, medium, low). Cultivated species were excluded from this consideration. *B. rapa* is partly cultivated and was therefore also excluded from the priority list. All species listed as endangered or near threatened on the IUCN Red List were assigned to the highest priority.

For all other species, the considerations included how many accessions from the natural occurrence countries are maintained in European genebanks. As already noted in the introduction, a major difficulty in this context is that for decades there has been controversy about the minimum number of accessions required to maintain the natural diversity of a species. Maxted et al. (2020) summarises these discussions very well. In addition to the existing literature, many years of experience from practical genebank work were therefore also taken into account when drawing up the priority list.

Furthermore, the predicted future reduction of the natural distribution ranges was included in the considerations. Based on the fact that human-induced global warming has increased at an unprecedented rate within the last ten years (Forster et al., 2023), we used the niche modelling results of the RCP 8.5 scenario and also assumed that no species migration takes place.

The procedure is described in detail in the **Supplementary File** “Priority list creation.pdf”, the creation of the priority list in **Supplementary Table 2**. The results are summarised in **Table 3**, which lists species from the rapeseed gene pool that should be collected with priority in their natural occurrence countries.

**Table 3:** Species from the rapeseed gene pool that should be collected with priority in their natural occurrence areas.

Taxon	Priority for collecting
*Brassica deserti* Danin & Hedge	high priority
*Brassica desnottesii* Emb. & Maire	high priority
*Brassica dimorpha* Coss. & Durieu	high priority
*Brassica elongata* Ehrh.	high priority
*Brassica fruticulosa* Cirillo	high priority
*Brassica gravinae* Ten.	high priority
*Brassica hilarionis* Post	high priority
*Brassica insularis* Moris	high priority
*Brassica maurorum* Durieu	high priority
*Brassica montana* Pourr.	high priority
*Brassica repanda* (Willd.) DC.	high priority
*Brassica souliei* Batt. subsp. *amplexicaulis* (Desf.) Greuter & Burdet	high priority
*Brassica souliei* Batt. subsp. *souliei* Batt.	high priority
*Descurainia sophia* (L.) Webb ex Prantl	high priority
*Diplotaxis siifolia* Kunze	high priority
*Enarthrocarpus lyratus* (Forssk.) DC.	high priority
*Erucastrum gallicum* (Willd.) O. E. Schulz	high priority
*Rorippa islandica* (Oeder) Borb	high priority
*Brassica bourgeaui* (Webb ex Christ) Kuntze	medium priority
*Brassica cretica* Lam.	medium priority
*Brassica incana* Ten.	medium priority
*Brassica nigra* (L.) W. D. J. Koch	medium priority
*Capsella bursa-pastoris* (L.) Medik.	medium priority
*Diplotaxis acris* (Forsk.) Boiss.	medium priority
*Diplotaxis catholica* (L.) DC.	medium priority
*Diplotaxis erucoides* (L.) DC.	medium priority
*Diplotaxis harra* (Forssk.) Boiss.	medium priority
*Diplotaxis muralis* (L.) DC.	medium priority
*Diplotaxis tenuifolia* (L.) DC.	medium priority
*Diplotaxis viminea* (L.) DC.	medium priority
*Moricandia arvensis* (L.) DC.	medium priority
*Moricandia nitens* (Viv.) E. A. Durand & Barratte	medium priority
*Rapistrum rugosum* (L.) All.	medium priority
*Sinapis arvensis* L.	medium priority
*Sinapis pubescens* L.	medium priority
*Brassica tournefortii* Gouan	low priority
*Eruca vesicaria* (L.) Cav.	low priority
*Hirschfeldia incana* (L.) Lagr.-Foss.	low priority

As a result, 18 species of the rapeseed gene pool were assigned to the highest category of the priority list. Here, it is reasonable to target the natural areas of origin and to collect additional material to be maintained *ex situ* in genebanks.

Therefore, further conservation and collecting strategies need to be developed for Europe at large. It should be taken into account that, especially for wild species, *in situ* conservation can play an important additional role, but reliable *ex situ* conservation is essential in any case. Data on the endangerment status can be used as a supplement in this context, but is not sufficient for a variety of species. An important role in the development of collecting strategies is taken by the expected effects of climate change on the natural distribution areas of the rapeseed gene pool species, as predicted by niche modelling. This makes it possible to react to future changes and to adapt the collection development of the genebanks accordingly.

## 4 Conclusion

In this study, we analysed the rapeseed gene pool and investigated to what extent the different species are conserved in European genebanks and which gaps exist. This also included the natural distribution ranges and it was found that most species of the rapeseed gene pool are significantly underrepresented in European genebanks. In addition, a niche modelling approach was used to investigate how the natural ranges of these species are likely to change by the end of the century under the assumption of various climate change scenarios. In some cases, considerable changes in the natural distribution areas were predicted. In order to close the existing gaps, a priority list was proposed. In addition to collecting trips, which are of course indispensable, sustainable conservation of CWRs requires a combination of *ex situ* and *in situ* efforts.

In general, various actions need to be taken to preserve CWRs, strategies are necessary to avoid loss of wild relatives of rapeseed: (1) Safeguarding the maintenance of all available CWR accessions in the genebanks in order to avoid further loss of material including regular regeneration and storage of a safety duplicate in Svalbard Global Seed Vault (2) Planning of collecting missions to increase the number of CWRs in genebanks for *ex situ* conservation. This should follow the priority list starting with the high priority species. (3) Undertaking also *in situ* conservation to increase the number of individuals in wild populations. (4) Protecting of natural habitats to prevent extinction. (5) Based on the niche modelling monitoring of the natural habitats. In case of loss due to climate change programmes for recolonisation or creation of new habitats.

## Data availability statement

The datasets used for the niche modelling as well as the detailed results were published in an online repository. They are accessible by the DOI: 10.5281/zenodo.8081795.

## Author contributions

SW coordinated the draft; SW, RH, KK, MO, RT and UL conceived and wrote the manuscript. All authors contributed to the article and approved the submitted version.

## Footnotes

1 The supplementary file “Niche modelling data.zip” is available from Zenodo by the DOI 10.5281/zenodo.8081795.
