# Supplementary material for: Analysis of gaps in rapeseed (Brassica napus L.) collections in European genebanks
Source: Front Plant Sci. 2023 Oct 9;14:1244467. doi: 10.3389/fpls.2023.1244467 (PMC10591083; doi:10.3389/fpls.2023.1244467)
Supplement: Supplementary file 3 [file DataSheet_1.pdf]

Compilation of a priority list:

The following describes the procedure used to create the priority list for targeted collection. As a result, three groups of highest, medium and low priority were created.

- Step 1: Exclusion of cultivated species
  - Cultivated species are not included in the priority list.
  - Species whose natural range is outside the study area are also excluded.
- Step 2: Inclusion of Red List status
  - All species listed as endangered or near threatening are assigned the highest priority.
  - The following steps are thus omitted for these species.
- Step 3: Comparison of the species with the number of accessions from the respective natural distribution areas.
  - As there is no clear consensus in the literature on the minimum number of accessions required to maintain natural diversity, a division is made into three groups, each of which is assigned a priority score between 1 (low) and 3 (high).
    - High (score 3): <= 50 accessions per species
    - Medium (score 2): <= 100 accessions per species
    - Low (score 1): <= 150 accessions per species
  - Species represented by larger numbers of accessions are not considered further here (score 0).
- Step 4: Consideration of predicted changes in the natural range.
  - We follow a conservative approach here and assume the pessimistic scenario RCP 8.5. Furthermore, it is assumed that no migration of species takes place.
  - Here, too, the species are divided into three groups, each of which is assigned a priority score between 1 (low) and 3 (high).
    - High (score 3): Expected reduction of the distribution area by >= 75%.
    - Medium (score 2): Expected reduction of the distribution area by >= 50%.
    - Low (score 1): Expected reduction of the distribution area by >= 25%.
  - Species for which a smaller reduction in range was predicted are not considered further here (score 0).
  - Those species for which no modelling could be carried out due to lack of data are assigned to the highest category (score 3). If no sufficient occurrence data are available, it must be assumed that the natural occurrences are very low.
- Step 5: Calculation of the total score and assignment of priority
  - The scores given in steps 3 and 4 are added up per species (equal weighting) and then result in the ranking of this species on the priority list:
    - 5-6 points: highest priority
    - 3-4 points: medium priority
    - 1-2 points: lowest priority
  - All other species are not considered for the priority list.
